# Impact of documentation on interpretation of administrative data

**DOI:** 10.1186/s13054-016-1186-8

**Published:** 2016-01-15

**Authors:** Sam Antonios

**Affiliations:** Department of Internal Medicine, Via Christi Hospitals, 929 N. St Francis, Wichita, KS 67214 USA

I read with great interest the recent article by Rhee et al. (“Improving documentation and coding for acute organ dysfunction biases estimates of changing sepsis severity and burden: a retrospective study”) in *Critical Care* [1]. The sensitivity of coding for acute organ dysfunction is increasing over time. More organ dysfunction codes are being captured. This change is the result of pressures on the health-care system in the United States. Across the nation, hospitals have created clinical documentation improvement programs to adjust for the previously noted poor documentation by physicians. Although the primary objective was financial, it was also noted that improved documentation leads to better public reporting of quality-of-care indices and improved health outcomes research. Several documented interventions have shown that specific education and training have increased the documentation of complication code (CC) and major complication code (MCC) capture rates for inpatients. Observed mortality did not change, but expected mortality increased, resulting in a decrease in median mortality index [2, 3]. This change in documentation practices may explain the raw mortality difference noted between European and US hospitals [4]. Further complicating the data will be the sepsis coding change that will occur now that ICD-10-CM (International Classification of Diseases, Tenth Revision, Clinical Modification) is the official coding classification. According to the ICD-10-CM Official Guidelines for Coding and Reporting, there is no longer a code for systemic inflammatory response syndrome (SIRS) resulting from infectious source. This can potentially affect the traditional SIRS-based definition of sepsis, particularly in cases in which other specific organ infections are documented. Severe sepsis or sepsis with an organ dysfunction will continue to require a minimum of two codes. For cases of septic shock, the code for the underlying systemic infection is required to be sequenced first. The heterogeneity of sepsis diagnosis-related groups, due either to capture of organ dysfunction or to coding of sepsis itself, should not be ignored. Administrative data are not reliable in recording a disease that includes a spectrum, such as sepsis [5]. This creates the need for more homogenous cohorts of patients within the administrative data, so that meaningful analysis can be performed more reliably, but also for a refinement of the risk adjustment methodologies that would make them more longitudinal.

## Abbreviations

ICD-10-CM: International Classification of Diseases, Tenth Revision, Clinical Modification
SIRS: systemic inflammatory response syndrome
